# Comparative study of two common methods for the determination of total mercury in soil

**DOI:** 10.1098/rsos.240757

**Published:** 2025-02-12

**Authors:** Pinzhu Qin, Ziyu Cai, Cuilan Wei, Ying Guan, Bowen Li, Jiali Huang, Xiamin Cheng

**Affiliations:** ^1^ School of Environment and Ecology, Jiangsu Open University, Nanjing 210017, People’s Republic of China; ^2^ Jiangsu Engineering and Technology Centre for Ecological and Environmental Protection in Urban and Rural Water Environment Management and Low Carbon Development, Jiangsu Provincial Department of Ecology and Environment, Nanjing 210000, People’s Republic of China; ^3^ Institute of Advanced Synthesis, School of Chemistry and Molecular Engineering, Jiangsu National Synergetic Innovation Center for Advanced Materials, Nanjing Tech University (Nanjing Tech), Nanjing 211816, People’s Republic of China

**Keywords:** accuracy, atomic fluorescence method, cold atomic absorption spectrophotometry, precision, total mercury in soil

## Abstract

Cold atomic absorption spectrophotometry and atomic fluorescence methods were employed to ascertain the total mercury concentration in soil samples. A systematic comparison and analysis were conducted on the operational procedures, precision and detection limits of these methodologies. The standard curve for total mercury determination in soil using the atomic fluorescence method is *y* = 851.98*x*+16.771 (*R*^2^ = 0.9996, 0–2.5 µg l^−1^), with a detection limit of 0.0006 mg kg^−1^. This method demonstrates superior precision and accuracy not only for samples with high and medium concentrations but also for those with low concentrations. For the cold atomic absorption spectrophotometry method, the standard curve for total mercury determination is *y* = 0.0547*x* + 0.0047 (*R*^2^ = 0.9998, 0–15 µg l^−1^). For high-concentration samples, the curve is *y* = 0.0007*x* − 0.0003 (*R*^2^ = 0.9998, 0–600 µg l^−1^). The detection limit for this method is 0.06 μg kg^−1^, indicating better sensitivity and accuracy compared with the atomic fluorescence method. While the precision for low-concentration (0.017 ± 0.003 mg kg^−1^) samples is moderate, it is higher for medium (0.030 ± 0.003 mg kg^−1^) and high-concentration (0.590 ± 0.05 mg kg^−1^) samples. Overall, there is no significant difference between the two detection methods. However, the cold atomic absorption spectrophotometry method has the advantage of not requiring pre-treatment, making it more convenient and environment friendly.

## 1. Introduction

The soil environment, with its natural fertility, promotes plant growth and is the material basis for human survival and agricultural development. The quality of the soil environment has an essential impact on food production, food safety, groundwater quality and air quality [1]. Soil pollution mainly refers to the process of long-term accumulation of pollutants in the soil, of which heavy metal pollution has a greater impact on human health. Heavy metal pollutants cannot be degraded by soil microorganisms and are continuously enriched in living organisms through the food chain. They can even be transformed into more toxic methyl compounds, posing a threat to human health. Mercury stands out as one of the most toxic elements in heavy metal pollution, with soil acting as the largest reservoir of mercury within the ecosystem. Mercury in soil exists in various forms, including monomeric mercury, inorganic mercury and organic mercury. The presence of mercury in soil has two primary sources: anthropogenic and natural. Anthropogenic sources predominantly include wastewater, waste gas, waste residue and other substantial amounts of mercury-containing waste materials discharged from industrial activities. Additionally, agricultural activities such as irrigation and fertilization alter the soil composition and its redox potential, leading to variations in mercury content and its chemical cycling.

Second, improper disposal of solid wastes containing mercury in daily life, such as thermometers, batteries, fluorescent lamps and sphygmomanometers, contributes to increased mercury levels in the soil around landfills and other disposal sites. Natural sources mainly involve the dry and wet atmospheric deposition of airborne gaseous mercury into the soil, which is then enriched in the soil pore water through the adsorption of clay minerals and organic matter in the soil surface layer, resulting in higher soil mercury content. Climate change also facilitates the atmospheric–soil exchange of mercury and the release of mercury from permafrost, further altering soil mercury content. Moreover, the transport of mercury, influenced by various soil factors such as soil parent material, pH, organic matter, soil texture and vegetation cover, can modify the mercury content of soils.

Mercury is a global pollutant with substantial neurotoxicity and bioconcentration and is one of the internationally recognized pollutants. In recent years, along with industrialization and urbanization, soil mercury pollution has become more serious, and the harm caused by mercury has become increasingly significant. Mercury in soil mainly enters the human body through digestion and absorption, skin contact, respiration and other exposure pathways, jeopardizing human health [1]. Among the other exposure pathways, diet is the primary source (the contribution ratio is 61.2%–99.8%; [2]). The natural environment is adversely affected by mercury contamination, as the substance percolates through the soil into groundwater, significantly impairing its quality. Additionally, dust particles from the Earth’s surface ascend into the atmosphere, contributing to the decline in air quality (research indicates that China emits 565.5 mg of zero-valent mercury into the atmosphere annually from soil sources) [2]. Plants absorb and concentrate mercury, which damages their morphology and physiological processes, severely impacting their growth and productivity [3]. This contamination is a primary concern for environmental monitoring efforts.

Mercury in the soil is present in complex forms and is primarily an insoluble compound. It is stable in the gaseous state at room temperature and pressure, and mercury released from soil can be transported over long distances in the atmosphere [3] and is a global pollutant. In order to protect the environment and human health, the United Nations Environment Programme promulgated the Minamata Convention on Mercury, which sets out a series of measures for the release and disposal of mercury [2]. The control and prevention of soil mercury pollution are of great significance. In order to implement the Law of the People’s Republic of China on Environmental Protection, protect the quality of the soil environment and control the risk of soil pollution, the State has approved two risk control standards [4]. Among them, the risk control standards for mercury pollution are listed in tables 1 and 2.

**Table 1.** Soil environmental quality soil pollution risk control standards for agricultural land (unit: mg kg^−1^).

item	risk screening value		risk control value
	paddy fields (mercury)	other (mercury)	mercury (chemistry)
pH ≤ 5.5	0.5	1.3	2.0
5.5 < pH ≤ 6.5	0.5	1.8	2.5
6.5 < pH ≤ 7.5	0.6	2.4	4.0
pH > 7.5	1.0	3.4	6.0

**Table 2.** Soil environmental quality construction land soil pollution risk control standards (unit: mg kg^−1^).

item	risk screening value		risk control value	
	type I land	category II sites	type I land	category II sites
mercury (chemistry)	8	38	33	82

Several approaches could be used for mercury detection. The spectrophotometric method is used to determine mercury by analyzing the absorbance of highly sensitive staining reagents such as dithizone, rhodanine, triazene, and cationic dyes that react directly or indirectly with mercury to form colored complexes or associations [5]. Dithizone spectrophotometry (GB7469-87) was the standard method for the determination of mercury in the early days, but the operation steps were complicated, and the sensitivity and selectivity were not satisfactory. In recent years, continuous research and improvement have led to the development of methods such as the nano-silver sol catalytic oxidation spectrophotometric method for the rapid determination of mercury in water [6]. These methods offer several advantages, including simple operation, affordability, rapidity, and sensitivity. Moreover, they can meet the requirements for monitoring mercury emission standards. Atomic absorption spectroscopy is currently a commonly used method for mercury determination, which mainly relies on the absorption of mercury vapor at the characteristic wavelength of 253.7 nm. Cold atomic absorption can quickly and accurately analyse samples containing mercury at the level of 10^-3^ mg kg^−1^ (or 10^−3^ mg l^−1^), and has the advantages of high sensitivity and high accuracy [6]. Catalytic pyrolysis-cold atomic absorption spectrophotometry (CAPAS) does not require chemical pre-treatment, and has the advantages of high precision, low detection limit and strong applicability while realizing automated analysis. CAPAS, such as the advanced mercury analyser, trace mercury analyser or direct mercury analyser does not require chemical pre-treatment, and has the advantages of high precision, low detection limit and strong applicability while realizing automated analysis [7–9]. Graphite furnace atomic absorption spectrometry, flame atomic absorption spectrometry and cold-vapour atomic absorption spectrometry have high sensitivity and are suitable for the direct determination of traces of mercury and automatic analysis has also been achieved with continuous research [10–12]. Atomic fluorescence spectrometry uses a unique mercury hollow-cathode lamp to produce atomic fluorescence. The fluorescence intensity is proportional to the mercury content. It is a convenient, fast, accurate and sensitive analytical method, suitable for the analysis of trace mercury at the level of 10^–9^–10^–12^ g g^–1^, and is a valuable method for mercury measurement in different complex sample matrices [8,13–15]. Cold vapour-atomic fluorescence spectrometry is a simple and reliable method, which is one of the techniques worth promoting for mercury morphology analysis [16]. Atomic emission spectrometry (AES) has not been used much in the morphological analysis of mercury, mainly in inductively coupled plasma-AES (ICP-AES) and microwave-induced plasma emission spectrometry (MIPES). ICP-AES in the environment to determine the analysis of metal materials application is very significant, and has become one of the main ways to analyse inorganic elements in metal materials [17], but the cost of equipment is relatively high. Though the MIPES has a higher excitation capacity and better stability than ICP-AES, its need for vacuum devices makes the operation complex and increases the operating costs, and it is currently in the exploratory stage [18]. In addition to the analytical techniques described above, inductively coupled plasma–mass or optical emission spectrometry (ICP-MS or ICP-OES, respectively) is used for the analysis of mercury [19,20]. ICP-MS has the advantages of simplicity, speed, high accuracy and precision and a wide range of determinations, but the memory effect of mercury is too strong and is generally not selected [21]. Additionally, chromatography plays a vital role in the morphological analysis of mercury. It is characterized by good separation, continuous determination and high sensitivity. Gas chromatography (GC), liquid chromatography (LC) and reversed-phase liquid chromatography are commonly used to analyse the morphology of methylmercury, ethylmercury, phenylmercury and inorganic mercury in samples. In daily use, GC and LC are combined with microwave plasma, inductively coupled plasma emission and atomic absorption methods, which integrate the high separation performance of chromatography with the high selectivity and sensitivity of spectrometry. With the advantages of a wide linear range and low detection limit, it has become an effective means of analysing the morphology of organic mercury [22]. Finally, the neutron activation analysis method allows for the simultaneous analysis of multiple elements. However, compared with some non-nuclear analytical methods, it is relatively expensive. The requirement for radiation stability of mercury complicates the detection process, and it is necessary to isolate certain interfering elements when analysing low-content biological samples. Consequently, the testing procedure becomes complex, labour-intensive and time-consuming, which hampers its widespread adoption [23].

The main methods for the determination of mercury in soil include the atomic fluorescence method (GB/T 22105.1-2008), cold atomic absorption spectrophotometry (GB/T 17136-1997) and CAPAS (HJ 923-2017). Among them, the solid injection-cold atomic absorption method has high precision, low detection limit, strong applicability and long stability of the calibration curve, which can effectively shorten the detection cycle of mercury in soil. Lu *et al*. [24] used the cold atomic absorption method to determine and analyse the mercury content in soil. In addition, the microwave digestion-atomic fluorescence method is capable of meeting the requirements for environmental samples and is deemed suitable for promoting its application in soil environmental monitoring. Xu *et al*. [25] employed this method to determine and analyse mercury content in soil. This article examines the limit of detection, precision and accuracy of total mercury (TM) in soil using two different methods: water bath digestion-atomic fluorescence and CAPAS, following the standard method.

## 2. Material and methods

### 2.1. Reagents

High-purity argon (99.999%) and high-purity oxygen (≥99.999%) were purchased from Nanjing Special Gas Factory Co., Ltd (Nanjing, China). Hydrochloric acid (ρ(HCl) = 1.19 g ml^−1^) and nitric acid (ρ(HNO_3_) = 1.42 g ml^−1^) purchased from Nanjing Chemical Reagent Co., Ltd (Nanjing, China), were of guaranteed reagent grade. A standard mercury solution (no. B22030174, 1000 mg l^−1^, 1.0 mol l^−1^ HNO_3_) and four standard samples for quality control with concentration codes GBW 07405 (GSS-5), GBW07404 (GSS-4), GBW07408 (GSS-8) and GBW07455 (GSS-26) were obtained from Beijing Zhongke Quality Inspection Biotechnology Co., Ltd (Beijing, China). Unless otherwise stated, all other reagents were of guaranteed reagent grade and used without purification or treatment. Ultrapure water was used throughout.

### 2.2. Sample pre-treatment

For the water bath digestion-atomic fluorescence method, we weighed out 0.2–1.0 g (accurate to 0.001 g) of a 100 mesh air-dried sample into a stoppered cuvette, moistened with a small amount of water, added 10 ml of (1+1) aqua regia, sealed with a stopper and shaken thoroughly. Subsequently, we dissolved the sample in a boiling water bath for 2 h, removed it and allowed it to cool. A preservation solution was added, diluted with diluent to the desired volume, shaken well and diluted further and the upper clear liquid layer was taken for measurement.

For CAPAS, we weighed 0.1 g to 0.5 g (accurate to 0.0001 g) of a 100 mesh air-dried sample into a sample boat (preferably a nickel boat) for analysis.

### 2.3. Catalytic pyrolysis-cold atomic absorption spectrophotometry

**Figure 1.**
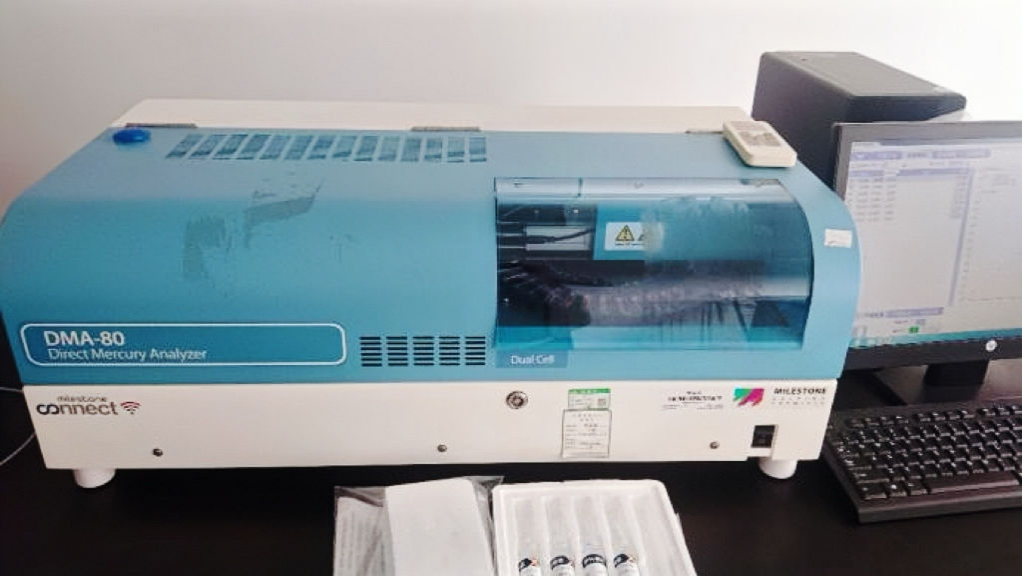
DMA-80 direct mercury meter.

After the sample is introduced into the combustion catalytic furnace, a series of processes including drying, thermal decomposition and catalytic reactions occur. These processes convert various forms of mercury into elemental mercury, which then enters the amalgamation tube to form gold amalgam. The amalgamation tube is rapidly heated to release the mercury from the gold amalgam in vapour form. This vapour, carried by the carrier gas, is directed into the cold atomic absorption spectrophotometer. Here, the mercury vapour absorbs the characteristic spectral line of 253.7 nm, and the intensity of this absorption is directly proportional to the concentration of mercury in a certain concentration range. A DMA-80 Direct Mercury Measuring Instrument was used for this process (figure 1), with the following instrument conditions: temperature set at 20 ± 5°C, humidity maintained between 20% and 70% and a carrier gas flow rate of 200 ml min^−1^.

### 2.4. Water bath digestion-atomic fluorescence method

**Figure 2.**
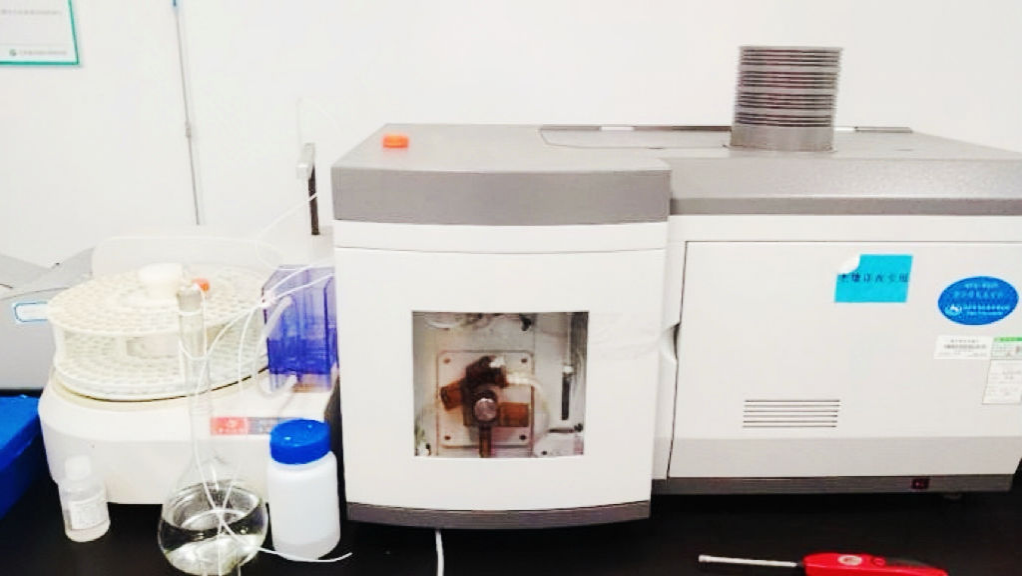
Model AFS-9320 fully automated six-lamp double sequential injection atomic fluorescence photometer.

The soil sample was digested in a water bath using a nitric acid and hydrochloric acid mixed reagent and then placed in an atomic fluorescence analyser, in which the elemental mercury in the potassium borohydride solution or sodium borohydride solution under the action of reduction, generated mercury atomic vapours. These gases are sent by the carrier gas (argon) to the argon–hydrogen flame to form the basal state atoms; atomic fluorescence intensity is proportional to the elemental content of the specimen in the special mercury lamp emitting light under the excitation of atomic fluorescence [26]. A model AFS-9320 Fully Automatic Six-Lamp Double Sequential Injection Atomic Fluorescence Photometer (figure 2) was used, and the instrument conditions were set as: temperature maintained at 20 ± 5°C, humidity ranging from 20% to 70%, a negative high voltage at 270 V, Hg lamp current of 60 mA, carrier gas flow at 400 ml min^−1^ and shielding gas flow at 800 ml min^−1^.

### 2.5. Methodology and criteria

#### 2.5.1. Catalytic pyrolysis-cold atomic absorption spectrophotometry

Experiments were conducted using CAPAS in accordance with ‘Determination of Mercury, Arsenic, Selenium, Bismuth and Antimony in Soil and Sediment Catalytic Pyrolysis/Cold Atomic Absorption Spectrophotometry’ (HJ 923-2017) [9] industry standards. Soil samples underwent processes such as drying, decomposition, ashing and reduction before being directly measured by a cold atomic absorption spectrometer. The drying temperature was 200°C and the pyrolysis temperature was 650°C.

#### 2.5.2. Water bath digestion-atomic fluorescence method

Elemental mercury was investigated under three commonly used pre-treatment methods, and the order of the determined elemental mercury content was as follows: microwave digestion＞direct determination ≈ water bath digestion within the error range of the guaranteed value for each sample [13]. Since the cold atomic absorption spectrophotometric method is used for direct determination, water bath digestion was chosen as the pretreatment method for the atomic fluorescence method according to the national standard ‘Determination of Total Mercury, Total Arsenic and Total Lead in Soil Quality Atomic Fluorescence Method’ (GB/T 22105.1-2008) [27], and industry standards of ‘Solid Waste Determination of Mercury, Arsenic, Selenium, Bismuth and Antimony by Microwave Disintegration/Atomic Fluorescence Method’ (HJ 702−2014), ‘Determination of Mercury, Arsenic, Selenium, Bismuth and Antimony in Soil and Sediment by Microwave Disintegration/Atomic Fluorescence Method’ (HJ 680-2013), ‘Leaching Toxicity of Solid Waste by Microwave Disintegration/Atomic Fluorescence Method’ (HJ 680-2013) and ‘Solid Waste Leaching Toxicity Leaching Method Sulfuric Acid Nitric Acid Method’ (HJ/T 299−2007). The soil samples were digested in aqua regia (1+1) in a boiling water bath for 2 h.

### 2.6. Methods of comparison

Analytical tests were conducted on blank samples, standard samples, quality control samples and actual samples to evaluate quality control indicators such as precision and accuracy, including standard deviation, relative error and significant difference:

- blank sample*:* a sample devoid of mercury, used as a procedural control in the determination of actual samples;
- standard samples: certified standard samples are commercially available;
- quality control samples: samples used to maintain quality standards in control samples; and
- actual samples: samples were obtained from the Jiangsu Institute of Environmental Science for soil testing.

### 2.7. Measurement

Mercury standard solution (with a concentration of 1000 mg l^−1^ in a 1% nitric acid solution) was used to establish the standard curve (no. B22030174; valid until 8 April 2023). Three standard samples with known concentrations of GBW 07405 (GSS-5), GBW07404 (GSS-4) and GBW07408 (GSS-8) along with one quality control sample, GBW07455 (GSS-26), were chosen to assess the precision and accuracy of various analytical methods. All standard substances were purchased from Beijing Zhongke Quality Inspection Biotechnology Co., Ltd.

## 3. Results

### 3.1. Linear range of the standard curve

#### 3.1.1. Catalytic pyrolysis-cold atomic absorption spectrophotometry

**Figure 3.**
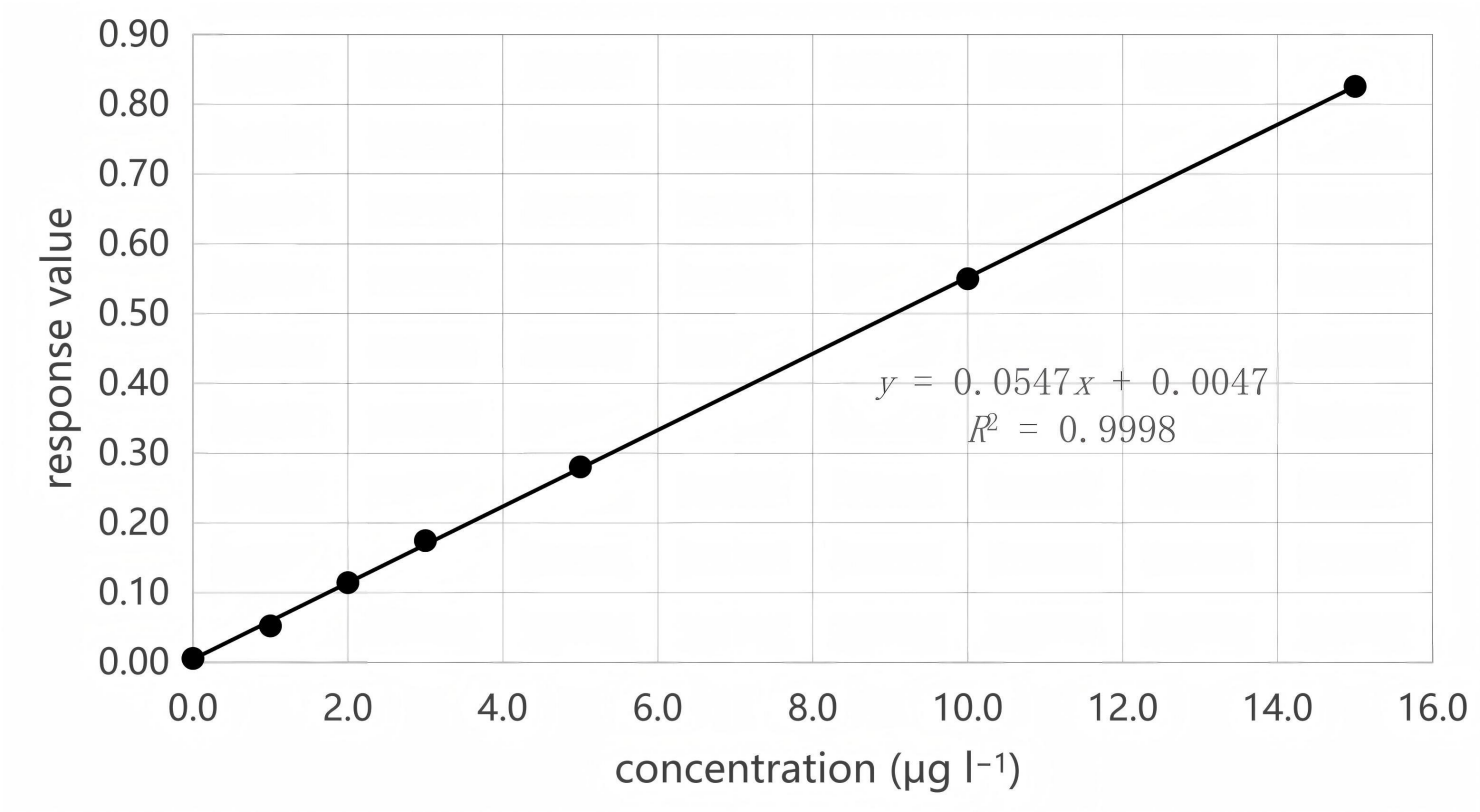
Plotting of standard curve for low concentration.

**Figure 4.**
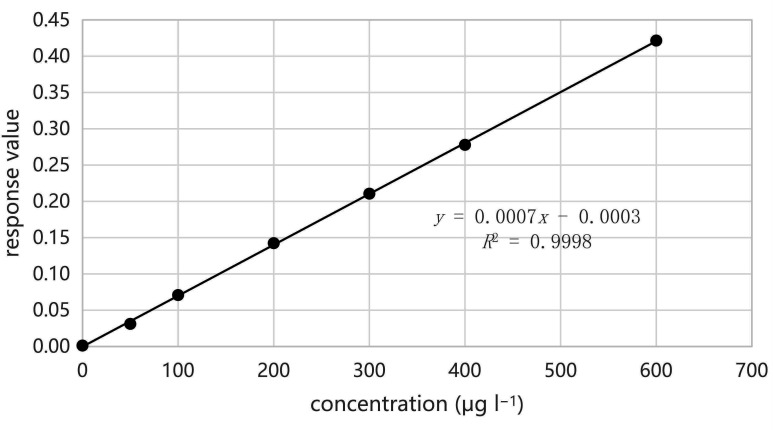
Plotting of a standard curve for high concentration.

The standard curve configurations are depicted in the previously reported reference [28]. Two standard curves were established: one for a low-concentration standard series solution (see figure 3) and another for a high-concentration standard series solution (see figure 4).

Linear range seven standard points were determined at low concentrations ranging from 0 to 15 µg l^−1^, and the resulting standard curve was *y* = 0.0547*x *+ 0.0047, with an *R*^2^ value of 0.9998. Seven standard points were determined at high concentrations ranging from 0 to 600 µg l^−1^, yielding a standard curve of *y* = 0.0007*x* − 0.0003, and an *R*^2^ value of 0.9998.

#### 3.1.2. Water bath heating-atomic fluorescence method

**Figure 5.**
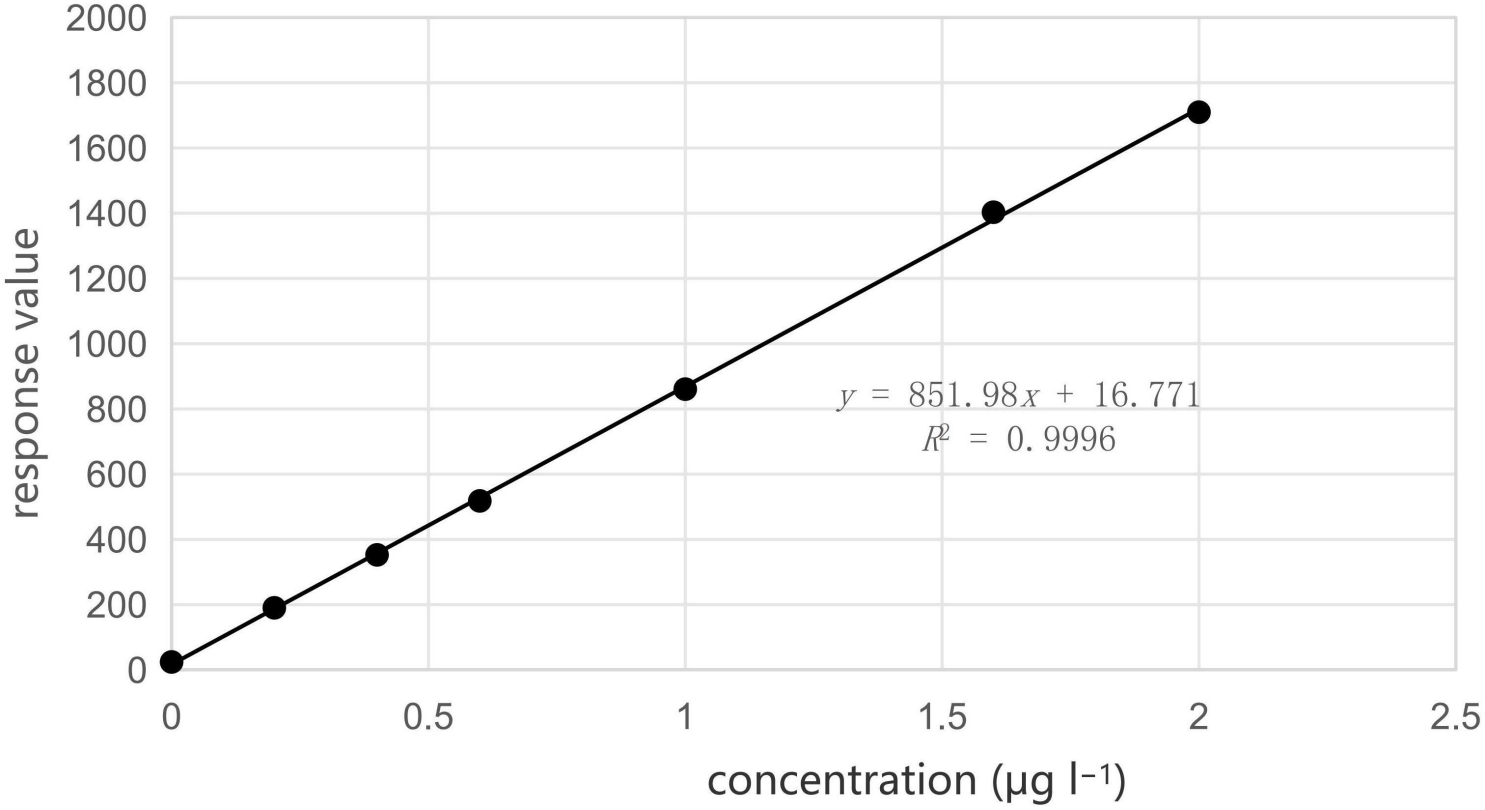
Plotting of the standard curve.

See [29] for standard curve configuration. The standard series of solutions were prepared (refer to figure 5 for outcomes). The linear range was established between 0 and 2.5 µg l^−1^ for seven standard points, and the resulting standard curve was *y* = 851.98*x *+ 16.771, *R*^2^ = 0.9996.

## 4. Discussion

### 4.1. Method detection limits

#### 4.1.1. Catalytic pyrolysis-cold atomic absorption spectrophotometer

The low-concentration spike of the samples was replicated seven times, and the standard deviations were calculated to determine the detection limits (results are presented in table 3).

**Table 3.** Catalytic pyrolysis-cold atomic absorption spectrophotometry results for low-concentration spiked determinations. (MDL=3.143S*V/M=3.143*0.021*0.01/0.1= 0.06 (ug kg^−1^); note: *S* is the standard deviation of the LC spiking of seven samples, MDL: Method Detection Limit).

elemental	measurements							MDL
mercury (mg kg^−1^)	0.0242	0.0211	0.0220	0.0240	0.0211	0.0212	0.0179	0.06

#### 4.1.2. Water bath heating-atomic fluorescence method

The low-concentration spike of the samples was replicated seven times, and the standard deviations were calculated to determine the detection limits (results are presented in table 4).

**Table 4:** . Results of low-concentration spiking determination by water bath heating-atomic fluorescence method. (MDL=3.143S*V/M=3.143*0.0012*0.05/0.3= 0.0006 (mg kg^−1^); note: *S* is the standard deviation of the LC spiking of seven samples.)

elemental	measurements							MDL
mercury (mg kg^−1^)	0.057	0.056	0.058	0.058	0.055	0.058	0.057	0.0006

### 4.2. Precision

#### 4.2.1. Catalytic pyrolysis-cold atomic absorption spectrophotometry

Seven determinations for each of the four standard samples with varying concentrations (GSS-4, GSS-5, GSS-8 and GSS-26) were analysed to assess precision, with results presented in table 5. The relative standard deviations (RSDs) of the catalytic pyrolysis-cold atomic absorption spectrophotometric method in determining low-concentration samples (GSS-8, GSS-26) were 9.1% and 5.3%, respectively, indicating an average level of precision. However, these RSDs were below 12.0%, satisfying the detection standard. For the middle- and high-concentration samples (GSS-4, GSS-5), the RSDs were 2.6% and 1.6%, respectively, demonstrating a high level of precision.

**Table 5.** Results of catalytic pyrolysis-cold atomic absorption spectrophotometry for four standard samples (mg kg^−1^). (RSD, relative standard deviation.)

serial number	1	2	3	4	5	6	7	RSD%
GSS-5	0.320	0.301	0.307	0.313	0.315	0.309	0.297	2.6
GSS-4	0.572	0.575	0.557	0.562	0.581	0.579	0.567	1.6
GSS-8	0.018	0.017	0.020	0.019	0.018	0.015	0.017	9.1
GSS-26	0.027	0.029	0.031	0.027	0.028	0.028	0.030	5.3

#### 4.2.2. Water bath heating-atomic fluorescence method

Seven determinations of each of the three standard samples with varying concentrations (GSS-4, GSS-8 and GSS-26) were analysed for precision, with the results presented in table 6.

**Table 6.** Determination of three standard samples by water bath heating-atomic fluorescence method (mg kg^−1^). (RSD, relative standard deviation.)

samples	1	2	3	4	5	6	7	RSD%
GSS-4	0.597	0.603	0.612	0.618	0.625	0.597	0.601	1.8
GSS-8	0.017	0.017	0.018	0.017	0.017	0.017	0.018	2.8
GSS-26	0.027	0.027	0.028	0.027	0.028	0.027	0.029	2.9

The RSDs of the catalytic pyrolysis-cold atomic absorption spectrophotometric method in determining low-concentration samples (GSS-8, GSS-26) were 9.1% and 5.3%, respectively, indicating average precision. However, these RSDs were below 12%, satisfying the detection criteria. For the middle and high-concentration samples (GSS-4, GSS-5), the RSDs were 2.6% and 1.6%, respectively, demonstrating high precision.

The RSDs in the determination of the three samples (GSS-4, GSS-8 and GSS-26) were 1.8%, 2.8% and 2.9% respectively, using the water bath heating-atomic fluorescence method. The refined densities were notably high in the assay of samples with high, medium and low concentrations.

### 4.3. Accuracy

#### 4.3.1. Catalytic pyrolysis-cold atomic absorption spectrophotometry

Seven determinations for each of the three standard samples with different concentrations (GSS-4, GSS-8 and GSS-26) were analysed for accuracy, with the results depicted in table 7.

**Table 7.** Determination of the accuracy of four standard samples by catalytic pyrolysis-cold atomic absorption spectrophotometry. (RE% is the average relative error of seven measurements; see the electronic supplementary material, table S4.)

samples	concentration (mg kg^−1^)	measured value (mg kg^−1^)	RE%
GSS-4	0.59 ± 0.05	0.570	−3.32
GSS-8	0.017 ± 0.003	0.018	4.20
GSS-26	0.030 ± 0.003	0.029	−4.76

#### 4.4.2. Water bath heating-atomic fluorescence method

Seven determinations for each of the three standard samples with varying concentrations (GSS-4, GSS-8 and GSS-26) were analysed for accuracy, as presented in table 8.

**Table 8.** Measured values of the accuracy of three standard samples by water bath heating-atomic fluorescence method. (RE% is the average relative error of seven measurements; see the electronic supplementary material, table S5.)

samples	concentration (mg kg^−1^)	measured value (mg kg^−1^)	RE%
GSS-4	0.59 ± 0.05	0.608	2.98
GSS-8	0.017 ± 0.003	0.017	1.68
GSS-26	0.030 ± 0.003	0.028	−8.10

**Figure 6.**
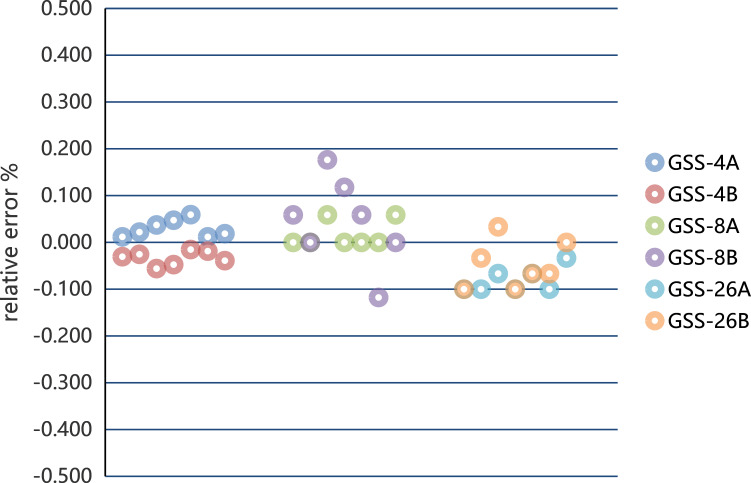
Scatter plot of RE of three standard samples determined by two methods (A, atomic absorption spectrophotometry; B, cold atomic absorption spectrophotometry).

A scatter plot of relative error (RE) for three standard samples determined by two methods are shown in figure 6. Statistical analysis of the data indicated that the correlation between RE and concentration was not significant, and the difference in RE between the two methods was essentially not significant, with RE ≤ ± 5%.

### 4.5. Significant differences

Atomic fluorescence and cold atomic absorption spectrophotometry were employed to ascertain the mercury content within the GSS-8 soil quality control samples, with the outcomes depicted in table 8. It was determined that the confidence level was *p* = 0.95, with *t*_0.05, 6_ = 2.24 at *f* = 6, and *t* = 1.43 < *t*_0.05, 6_ for the atomic fluorescence method, and *p* = 0.95, *t*_0.05, 6_ = 2.24 at *f* = 6, *t* = 1.09 < *t*_0.05, 6_ for cold atomic absorption spectrophotometry, respectively. This indicated that there was no significant difference compared with the mean μ. A statistical analysis was conducted on the two methods, with *p* = 0.95 and *f* = 12, *t*_0.05, 20_=2.09, and *t* = 2.03 < *t*=_0.05, 20_, further demonstrating that there was no significant difference in the average measurement results between the two methods.

**Table 9.** Experimental results of the two methods (mg kg^−1^).

serial number	atomic fluorescence method	cold atomic absorption spectrophotometry
1	0.017	0.018
2	0.017	0.017
3	0.018	0.020
4	0.017	0.019
5	0.017	0.018
6	0.017	0.015
7	0.018	0.017

## 5. Conclusion

The soil environment is intrinsically linked to human well-being, forming the very foundation upon which our lives and the health of our planet depend. Its quality has a profound and enduring impact on human health, influencing everything from the nutritional content of our food to the prevalence of environmental diseases. Moreover, the ecological environment, which encompasses the complex web of life that sustains our planet, is also deeply affected by the quality of the soil over the long term. The mercury content within the soil is a crucial parameter to monitor, as mercury is a potent neurotoxin that can accumulate in the food chain, posing significant risks to both human health and wildlife.

Given the importance of this parameter, a suitable analytical method is indispensable for accurately measuring mercury levels in the soil. The atomic fluorescence method, a sophisticated technique that excites atoms with ultraviolet light and measures the resulting fluorescence, has been shown to yield an excellent standard curve within the range of 0–2.5 µg l^−1^. This method exhibits a strong linear relationship, high precision and accuracy, making it a valuable tool in the environmental scientist’s arsenal. However, it is worth noting that the detection limit of the atomic fluorescence method is higher than that of the cold atomic absorption spectrophotometry, a technique that measures the amount of light absorbed by mercury vapour at a specific wavelength.

On the other hand, the cold atomic absorption spectrophotometric method, which also excels in the detection of mercury, has achieved good standard curves in the ranges of 0–15 and 0–600 µg l^−1^. This method boasts excellent linearity, a lower detection limit and high accuracy, which are critical for ensuring that even the smallest traces of mercury can be detected and quantified. Despite these advantages, the precision of the cold atomic absorption spectrophotometric method is marginally inferior to that of the atomic fluorescence method when analysing low-concentration samples.

In comparing the two methods, there is no significant disparity in their overall effectiveness for determining TM in the soil environment. Both methods are capable of providing reliable data that can inform policy decisions and guide remediation efforts. However, in the context of this particular experiment, the cold atomic absorption spectrophotometric method has proved to be more convenient and eco-friendly than the atomic fluorescence method. This is owing to its lower energy requirements and the fact that it does not necessitate the use of potentially hazardous chemicals, making it a more sustainable choice for environmental monitoring.

## Data Availability

All used data are included in the electronic supplementary material [30].
